# Proteomic and Phosphoproteomic Profiling of High-Fat Diet-Induced Hepatic Steatosis in Mice

**DOI:** 10.3390/nu18213587

**Published:** 2026-10-29

**Authors:** Lei Feng, Ziye Zhang, Zilu Wang, Zhiyong Hu, Ruina Zhai

**Affiliations:** Key Laboratory of Efficient Utilization of Non-Grain Feed Resources (Co-Construction by Ministry and Province), Ministry of Agriculture and Rural Affairs, Shandong Provincial Key Laboratory of Animal Nutrition and Efficient Feeding, College of Animal Science and Technology, Shandong Agricultural University, Tai’an 271017, China

**Keywords:** high-fat diet, hepatic steatosis, proteomics, phosphoproteomics, lipid metabolism

## Abstract

Background: Long-term high-fat diet (HFD) consumption induces hepatic lipid accumulation and metabolic dysregulation, but its integrated effects on the hepatic proteome and phosphoproteome remain incompletely understood. This study investigated HFD-induced hepatic molecular alterations using quantitative proteomics and phosphoproteomics. Methods: Eight-week-old male C57BL/6J mice were fed a control diet (CON) or HFD for 16 weeks, followed by phenotypic assessment, serum redox assays, and hepatic proteomic and phosphoproteomic analyses. Results: HFD-fed mice exhibited increased body weight, fat mass, liver weight, and marked hepatic lipid accumulation. Blood glucose levels and total pyruvate tolerance test area under the curve (AUC) were increased, whereas baseline-adjusted incremental AUC did not differ significantly. Serum malondialdehyde levels were elevated, whereas glutathione peroxidase activity was reduced, indicating impaired glucose homeostasis and redox balance. Proteomic analysis identified 15 differentially expressed proteins (4 upregulated and 11 downregulated), associated with lipid metabolism, biological oxidation, and xenobiotic metabolism. Gene set enrichment analysis identified significant positive enrichment of Class I peroxisomal membrane protein import and significant negative enrichment of cholesterol biosynthesis, gluconeogenesis, and xenobiotics. Phosphoproteomic analysis identified 8593 phosphoproteins and 28,180 phosphorylation sites, including 2045 differentially phosphorylated sites (1044 increased and 1001 decreased). Enrichment and exploratory network analyses implicated mitophagy, glucose metabolism, mTOR signaling, autophagy, oxidative phosphorylation, and cytoskeletal organization. Conclusions: Long-term HFD feeding induced obesity, glucose dysregulation, hepatic steatosis, and redox imbalance. Fatty acid oxidation and peroxisomal abundance patterns may reflect metabolic adaptation to lipid loading, while phosphosite abundance changes link nutrient sensing and autophagy regulation to the hepatic response.

## 1. Introduction

Chronic nutrient excess is a major driver of obesity and related metabolic diseases. A high-fat diet (HFD) disrupts systemic energy homeostasis [1] and induces a range of metabolic abnormalities, including obesity, insulin resistance, dyslipidemia, and hepatic lipid accumulation [2,3]. The liver is a central organ in the regulation of systemic glucose and lipid homeostasis, maintaining energy balance by coordinating fatty acid uptake and oxidation, lipid synthesis and export, glycolysis, and gluconeogenesis. Long-term HFD consumption disrupts this balance, leading to abnormal hepatic lipid accumulation accompanied by oxidative stress, mitochondrial dysfunction, inflammation, and cellular stress, thereby promoting the development and progression of metabolic dysfunction-associated steatotic liver disease (MASLD) [4].

Hepatic lipid homeostasis depends on the dynamic balance among fatty acid uptake, de novo lipogenesis, fatty acid oxidation, triglyceride synthesis, and very-low-density lipoprotein secretion [5]. Hepatic triglycerides in fatty liver do not originate from a single pathway but are derived from circulating free fatty acids, de novo lipogenesis, and dietary lipids [6]. Sustained HFD consumption increases both the supply of exogenous fatty acids and the flux of adipose tissue-derived fatty acids. It may also alter the expression or activity of metabolic enzymes such as ACLY, ACC, FASN, and CPT1, progressively disrupting the balance among lipid synthesis, degradation, and export [7]. As the lipid burden increases, mitochondria and peroxisomes may compensate by enhancing their capacity for fatty acid oxidation. However, when this compensatory response is insufficient to process the excess substrates, incomplete fatty acid oxidation, reactive oxygen species production, and disruption of cellular redox homeostasis may further aggravate hepatocellular stress. Therefore, HFD-induced hepatic lipid accumulation cannot be attributed simply to a single anabolic or catabolic pathway; rather, it likely results from the collective dysregulation of lipid uptake, utilization, storage, and export networks.

HFD-induced hepatic metabolic abnormalities involve extensive remodeling of complex molecular networks. As proteins are the principal effectors of cellular functions, changes in their abundance can directly reflect the metabolic and functional state of the liver. Mass spectrometry-based quantitative proteomics provides a powerful approach for systematically characterizing HFD-induced molecular alterations in the liver. Previous studies have shown that long-term HFD consumption markedly alters the hepatic proteomic profile in mice, with differentially expressed proteins broadly involved in lipid metabolism, fatty acid oxidation, and other metabolic processes [1]. Moreover, quantitative proteomic analysis of mice with HFD-induced fatty liver identified numerous differentially expressed proteins closely associated with lipid metabolism, as well as potential transcription factors involved in its regulation. These findings demonstrate that proteomics can provide new molecular insights into HFD-induced hepatic metabolic remodeling [5]. These findings indicate that proteomics can complement conventional biochemical measurements by identifying changes across multiple metabolic processes. However, protein abundance represents only one level of regulation. Even in the absence of substantial changes in total abundance, many metabolic enzymes and signaling proteins can undergo rapid alterations in activity, stability, subcellular localization, or protein–protein interactions through post-translational modifications. Therefore, changes in total protein abundance alone are insufficient to fully explain the alterations in hepatic metabolic function induced by long-term HFD consumption.

Protein phosphorylation is an important reversible modification involved in cellular responses to hormones, nutrients, and stress, and its dynamic balance is maintained through the coordinated actions of protein kinases and phosphatases [8]. Previous studies have shown that high-fat diet-induced hepatic metabolic dysfunction is accompanied not only by changes in protein abundance but also by extensive remodeling of protein phosphorylation signaling networks involving metabolic enzymes and receptor-mediated signal transduction, indicating that lipid overload can simultaneously affect protein expression and post-translational regulation [9]. Integrated proteomic, phosphoproteomic, and lipidomic analyses of the livers of HFD-fed mice have further demonstrated that differentially phosphorylated proteins are closely associated with fatty acid metabolism, insulin signaling, and lipid-mediated cellular signaling [10]. Compared with the analysis of protein abundance alone, phosphorylation-site analysis can capture dynamic alterations in nutrient-sensing and metabolic signaling networks, thereby helping to explain discrepancies between protein abundance and functional state.

Within phosphorylation networks associated with hepatic lipid homeostasis, AMP-activated protein kinase (AMPK), mechanistic target of rapamycin complex 1 (mTORC1), and UNC-51-like kinase 1 (ULK1) serve as key regulatory nodes linking cellular energy status to autophagy initiation. Under energy-deficient conditions, AMPK promotes autophagy by directly phosphorylating ULK1. Conversely, under nutrient-replete conditions, mTORC1 phosphorylates ULK1 and restricts autophagy initiation [11]. Autophagy mediates the clearance of damaged proteins and organelles. Lipophagy, a selective form of autophagy targeting lipid droplets, delivers lipid droplets to lysosomes for degradation and fatty acid release. Inhibition of autophagy in hepatocytes increases hepatic triglyceride storage and lipid droplet accumulation [12], whereas obesity-associated impairment of hepatic autophagy exacerbates endoplasmic reticulum stress and compromises insulin signaling [13]. Thus, AMPK–mTORC1–ULK1-mediated regulation of autophagy may represent an important link between nutrient excess, lipid droplet turnover, and organelle quality control [14,15,16].

Accordingly, this study used C57BL/6J mice fed a long-term HFD as the experimental model, with normal chow-fed mice serving as controls. Quantitative proteomics and phosphoproteomics were employed to systematically characterize HFD-induced alterations in hepatic protein abundance and phosphorylation. By integrating differentially expressed proteins, differentially phosphorylated sites, and associated biological pathways, this study investigated HFD-induced remodeling of hepatic metabolic and signaling networks at both the protein abundance and post-translational modification levels, providing a basis for further elucidating the molecular mechanisms underlying hepatic metabolic abnormalities caused by chronic nutrient excess.

## 2. Materials and Methods

### 2.1. Experimental Animals and Study Design

A total of 24 healthy eight-week-old specific pathogen-free (SPF) male C57BL/6J mice with similar body weights were obtained from Vital River Laboratory Animal Technology Co., Ltd. (Beijing, China). These animal inclusion criteria were established a priori. Mice were randomly assigned to a control group (CON) or a high-fat diet group (HFD) using a random number table, with 12 mice per group. Mice in the CON group were fed a normal chow diet, whereas those in the HFD group were fed a high-fat diet providing 60% of total energy from fat for 16 consecutive weeks. Food and water were provided ad libitum, and body weight was recorded weekly. No animals were excluded during the experiment, and no data points were excluded from the analyses.

All mice were housed in an SPF animal facility maintained at 23 ± 2 °C and 50 ± 10% relative humidity under a 12 h light/12 h dark cycle. Mice were acclimatised for one week before the experiment, and their health and behaviour were monitored daily. Animals were handled gently, and unnecessary handling and restraint were minimised to reduce distress. Isoflurane anaesthesia was used during terminal blood collection and tissue harvesting to minimise pain and distress.

Experimental procedures and measurements were performed by alternating between the CON and HFD groups, with the order of animals within each group randomised, and were completed within consistent time windows. Proteomic samples from both groups were analysed in the same analytical batch. All animal experimental protocols were approved by the Laboratory Animal Welfare and Animal Experiment Ethics Review Committee of Shandong Agricultural University (protocol code: SDAUA-2024-238; date of approval: 12 September 2024). Outcome measures included body weight, body fat mass, liver weight, blood glucose concentrations and the area under the curve during the intraperitoneal pyruvate tolerance test, hepatic histological changes and lipid accumulation, serum malondialdehyde levels and glutathione peroxidase activity, and hepatic protein and phosphorylation-site abundances. Serum malondialdehyde levels and glutathione peroxidase activity were measured using commercial assay kits (Shanghai Enzyme-linked Biotechnology Co., Ltd., Shanghai, China) according to the manufacturer’s instructions. The remaining measurement procedures and analytical criteria are described in the following sections.

### 2.2. Pyruvate Tolerance Test

Pyruvate serves as a substrate for gluconeogenesis; therefore, the increase in blood glucose following intraperitoneal pyruvate administration can be used to assess gluconeogenic capacity in mice. Sodium pyruvate was dissolved in physiological saline at 6 g/30 mL to prepare a 20% (*w*/*v*) solution, which was subsequently passed through a 0.22 μm filter. Six mice from each group were intraperitoneally injected with sodium pyruvate at 2 g/kg body weight, corresponding to an injection volume of 0.01 mL/g body weight. Mice were fasted for 16 h before the IPPTT. Fasting blood glucose was measured before injection, and blood samples were collected from the tail at 15, 30, 60, 90, and 120 min after injection to determine blood glucose concentrations. Baseline-adjusted incremental AUC (iAUC) was calculated for each mouse over 0–120 min using the trapezoidal rule after subtracting its own 0 min blood glucose concentration from all time points, retaining any areas below baseline as negative contributions.

### 2.3. Oil Red O Staining of Liver Sections

Fixed liver tissues were sequentially cryoprotected in 15%, 30%, and 40% sucrose solutions, embedded in optimal cutting temperature (OCT) compound, rapidly frozen, and sectioned at a thickness of 8 μm. The frozen sections were equilibrated to room temperature and air-dried before Oil Red O staining. A working solution was prepared by mixing saturated Oil Red O solution with distilled water at a ratio of 6:4. Sections were stained for 8–10 min in the dark, differentiated with 60% isopropanol, and counterstained with hematoxylin. After staining, the sections were mounted with glycerol gelatin, examined microscopically for hepatic lipid deposition, and imaged for subsequent analysis. Liver samples from three randomly selected mice per group were used for histological assessment. Three non-overlapping microscopic fields per mouse were randomly selected at the same magnification. All sections and corresponding images were coded, and assessors were blinded to group allocation throughout field selection and quantitative image analysis.

### 2.4. Measurement of Fat Mass

Mice in each group were anesthetized via isoflurane inhalation. Once stable anesthesia was achieved, the mice were positioned with their limbs and trunk naturally extended. Whole-body scans were performed using dual-energy X-ray absorptiometry (DXA) to determine fat mass. After scanning, the mice were allowed to recover in a temperature-controlled environment and were returned to their home cages after resuming normal activity.

### 2.5. Proteomic Analysis

Liver samples were placed in 2 mL grinding tubes containing precooled protein lysis buffer and one 6 mm steel bead and subjected to three cycles of wet grinding for 180 s each. The homogenates were then incubated at 95 °C and 1500 rpm for 5 min in a thermomixer, followed by centrifugation at 14,000× *g* for 10 min. The supernatants were collected, protein concentrations were determined using the bicinchoninic acid (BCA) assay, and protein quality was evaluated by sodium dodecyl sulfate–polyacrylamide gel electrophoresis (SDS–PAGE). An appropriate amount of each protein sample was adjusted to a final volume of 20 μL with sodium deoxycholate (SDC). After reduction and alkylation, the samples were incubated at 45 °C and 1000 rpm for 5 min. Proteolytic digestion reagents were subsequently added, and the samples were digested at 37 °C and 1000 rpm for 4 h to overnight. The resulting digests were acidified and centrifuged, after which the supernatants were collected, desalted using StageTips containing C18 disks (3M, St. Paul, MN, USA), and vacuum-dried. Peptide concentrations were measured using a NanoDrop One spectrophotometer (Thermo Fisher Scientific, Waltham, MA, USA).

Equal amounts of peptides were subjected to data-independent acquisition (DIA) analysis. Peptides were separated using a Vanquish Neo liquid chromatography system (Thermo Fisher Scientific, Waltham, MA, USA) equipped with a custom-packed analytical column (15 cm × 100 μm, 1.7 μm). Mobile phase A consisted of 2% acetonitrile and 0.1% formic acid in water, whereas mobile phase B consisted of 80% acetonitrile and 0.1% formic acid in water. The chromatographic run time was 8 min. Mass spectrometric analysis was performed using an Astral mass spectrometer (Thermo Fisher Scientific, Waltham, MA, USA) in positive-ion mode with an ion-source voltage of 2.2 kV. The MS and MS/MS scan ranges were 380–980 and 150–2000 *m*/*z*, respectively.

The DIA raw data were analyzed using Spectronaut™ 19 software and searched against the UniProt database. Trypsin/P was specified as the proteolytic enzyme, with up to two missed cleavages allowed. Oxidation of methionine [oxidation (M)] and acetylation of the protein *N*-terminus [acetyl (protein *N*-term)] were set as variable modifications, whereas carbamidomethylation of cysteine [carbamidomethyl (C)] was set as a fixed modification. The false discovery rates (FDRs) at both the protein and peptide levels were controlled at ≤1%, and protein quantification was performed using the MaxLFQ method.

### 2.6. Phosphoproteomic Analysis

Frozen liver samples were lysed in protein lysis buffer containing 8 M urea, 1% SDS, and phosphatase inhibitors. Samples were homogenized for three cycles of 40 s each using a tissue homogenizer and lysed on ice for 30 min, with vortexing for 5–10 s every 5 min. The lysates were subsequently centrifuged at 12,000× *g* for 30 min at 4 °C, and the supernatants were collected for protein quantification using the BCA assay. For each sample, 800 μg of protein was adjusted to a final triethylammonium bicarbonate (TEAB) concentration of 100 mM and sequentially reduced with 10 mM tris(2-carboxyethyl)phosphine (TCEP) and alkylated with 40 mM iodoacetamide. Proteins were precipitated by adding six volumes of precooled acetone and incubating at −20 °C for 4 h. After centrifugation at 10,000× *g* for 20 min, the protein pellets were collected and redissolved in 100 mM TEAB. Trypsin was added at an enzyme-to-protein mass ratio of 1:50, and digestion was performed overnight at 37 °C. The resulting peptides were desalted using HLB cartridges, vacuum-dried, and quantified.

Phosphopeptides were enriched using TiO_2_ magnetic beads. The entire desalted peptide preparation derived from 800 μg of starting protein per sample was resuspended in 100 μL of loading buffer (1 M glycolic acid, 80% acetonitrile, and 5% TFA; pH < 3) and incubated with pre-equilibrated beads for 1 h with rotation. The beads were washed once with loading buffer, three times with 80% acetonitrile/1% TFA, and twice with 10% acetonitrile/0.2% TFA. Bound phosphopeptides were eluted three times with 80 μL of 1% NH_4_OH for 15 min per elution with vortexing. The pooled eluates were acidified to approximately pH 3 using 20% TFA, lyophilized, desalted using StageTips containing C18 disks (3M, St. Paul, MN, USA), and lyophilized again. Enrichment efficiency was calculated as the number of phosphopeptide-spectrum matches (PSMs) divided by the total number of PSMs × 100%, ranging from 32.5% to 39.9% across the six samples.

Equal amounts of enriched peptides were analyzed by liquid chromatography–tandem mass spectrometry (LC–MS/MS) using a Vanquish Neo liquid chromatography system coupled to an Orbitrap Astral mass spectrometer (Thermo Fisher Scientific, Waltham, MA, USA). Peptide separation was performed on a C18 column (250 mm × 75 μm, 1.6 μm). Mobile phase A consisted of water containing 2% acetonitrile and 0.1% formic acid, whereas mobile phase B consisted of water containing 80% acetonitrile and 0.1% formic acid. The flow rate was 300 nL/min. Mass spectrometric data were acquired in positive-ion DIA mode with an ion-source voltage of 1.5 kV.

The DIA raw data were analyzed using Spectronaut™ 19 software. Trypsin/P was specified as the proteolytic enzyme, with up to two missed cleavages allowed. Oxidation of methionine [oxidation (M)], acetylation of the protein *N*-terminus [acetyl (protein *N*-term)], and phosphorylation of serine, threonine, and tyrosine [phospho (STY)] were set as variable modifications, whereas carbamidomethylation of cysteine [carbamidomethyl (C)] was set as a fixed modification. The false discovery rates (FDRs) at both the protein and peptide levels were controlled at ≤1%, and protein quantification was performed using the MaxLFQ method. Phosphorylation localization confidence was assessed using the PTMAssayProbability metric in Spectronaut, with a minimum threshold of 0.75. The precursor-level false discovery rate (FDR) was controlled at ≤1%. Only precursors meeting both criteria were included in phosphosite quantification.

### 2.7. Western Blot Analysis

Liver proteins were extracted using RIPA buffer containing protease and phosphatase inhibitors. After centrifugation at 12,000 rpm for 5 min at 4 °C, supernatant protein concentrations were determined using a BCA assay and equalized. Samples were mixed with loading buffer, heated at 100 °C for 5 min, and separated by SDS-PAGE using 6–20% FuturePAGE™ precast gels (110 V). Proteins were transferred onto methanol-activated 0.45 μm PVDF membranes at 200 mA for 1.5 h under cooled conditions using transfer buffer containing 20% ethanol.

Membranes were blocked with a protein-free rapid blocking solution at 37 °C for 30 min and incubated with primary antibodies against p-AKT (Ser473) and total AKT at 4 °C for 12 h. Following three 10 min washes with TBST, membranes were incubated with horseradish peroxidase-conjugated secondary antibodies at 4 °C for 4 h and washed again. Bands were visualized using enhanced chemiluminescence and quantified using ImageJ (version 1.54p). The p-AKT/AKT ratio was normalized to the mean value of the CON group. Six mice per group were analyzed.

### 2.8. Statistical Analysis

Unless otherwise specified, statistical analyses were performed using R version 4.5.0, and data are presented as the mean ± standard error of the mean (SEM). Comparisons between the CON and HFD groups were performed using a two-tailed independent-samples *t*-test. Blood glucose curves from the intraperitoneal pyruvate tolerance test (IPPTT) were analyzed using two-way repeated-measures analysis of variance, with time as the within-subject factor and dietary treatment as the between-subject factor. The Geisser–Greenhouse correction was applied to account for violations of sphericity, and Šídák’s multiple-comparisons test was used to assess between-group differences at each time point. Differences were considered statistically significant at *p* < 0.05. The iAUC values were compared between groups using a two-tailed unpaired Welch’s *t*-test. Graphs of mouse phenotypic data and the corresponding statistical results were generated using GraphPad Prism version 10.1.2.

Fold change (FC) was calculated as the ratio of the mean abundance of each protein or phosphorylation site in the HFD group to that in the CON group. Differential abundance analysis was performed using limma (version 3.66.0), and the resulting two-sided *p* values were adjusted using the Benjamini–Hochberg (BH) procedure separately across all tested proteins and all tested phosphorylation sites. For the global proteomic dataset, proteins with a BH-adjusted *p* value ≤ 0.05 and either FC ≥ 1.20 or FC ≤ 0.83 were defined as differentially abundant proteins; FC ≥ 1.20 indicated increased abundance, whereas FC ≤ 0.83 indicated decreased abundance. For the phosphoproteomic dataset, sites with a BH-adjusted *p* value ≤ 0.05 and either FC ≥ 1.50 or FC ≤ 0.67 were defined as differentially phosphorylated sites; FC ≥ 1.50 indicated increased phosphosite abundance, whereas FC ≤ 0.67 indicated decreased phosphosite abundance. Complete differential-testing results for all tested proteins and phosphorylation sites are provided in Tables S1 and S2, respectively.

For the supplementary parent-protein-adjusted analysis, phosphosites were matched to parent proteins by exact accession IDs. Within each sample, log_2_ parent-protein abundance was subtracted from log_2_ phosphosite abundance. The 7565 matched phosphosites were analyzed using limma with BH correction; unmatched sites were excluded. Differential sites were defined by an adjusted *p* value ≤ 0.05 and an HFD/CON fold change ≥ 1.50 or ≤0.67. Complete results are provided in Table S3.

Gene Ontology (GO) enrichment analysis was performed for the BH-significant differentially abundant proteins and proteins corresponding to the BH-significant differentially phosphorylated sites across the biological process, cellular component, and molecular function categories. Pathway enrichment analysis was conducted using the Kyoto Encyclopedia of Genes and Genomes (KEGG) database. Reactome pathway enrichment analysis was additionally performed for differentially abundant proteins. Enrichment *p* values were adjusted using the BH procedure, and terms or pathways with an adjusted *p* value < 0.05 were considered significantly enriched.

Gene set enrichment analysis (GSEA) was performed using all quantified proteins. With the CON group as the reference, proteins were ranked using the Signal2Noise metric. Reactome pathway gene sets were obtained from the mouse collection in the Molecular Signatures Database (MSigDB), and only gene sets containing 15–500 genes were included. Normalized enrichment scores (NESs) were used to describe the direction of gene set enrichment. Statistical significance was defined as an FDR *q* value < 0.05; positive or negative NES values that did not meet this threshold were interpreted as exploratory directional patterns.

For the global proteomic dataset, an exploratory PPI analysis was performed using the original protein set selected with an unadjusted two-sided *t*-test *p* value ≤ 0.05 and either FC ≥ 1.20 or FC ≤ 0.83. For the phosphoproteomic dataset, an exploratory PPI analysis was performed using proteins harboring at least one phosphosite selected with an unadjusted Student’s *t*-test *p* value ≤ 0.05 and either FC ≥ 1.50 or FC ≤ 0.67. Both exploratory PPI analyses were conducted separately from the BH-adjusted principal discovery analyses. Protein–protein interaction information was obtained from the STRING database (version 11.5), with the species restricted to *Mus musculus*. Networks were constructed using the 150 highest-ranked protein–protein interactions based on the STRING combined interaction score and visualized using Cytoscape version 3.10.4. The networks were further organized according to network connectivity and the principal biological processes investigated in this study, and the major functional modules were classified based on enrichment results and protein functional annotations.

## 3. Results

### 3.1. High-Fat Diet-Induced Obesity and Hepatic Steatosis

**Figure 1:**
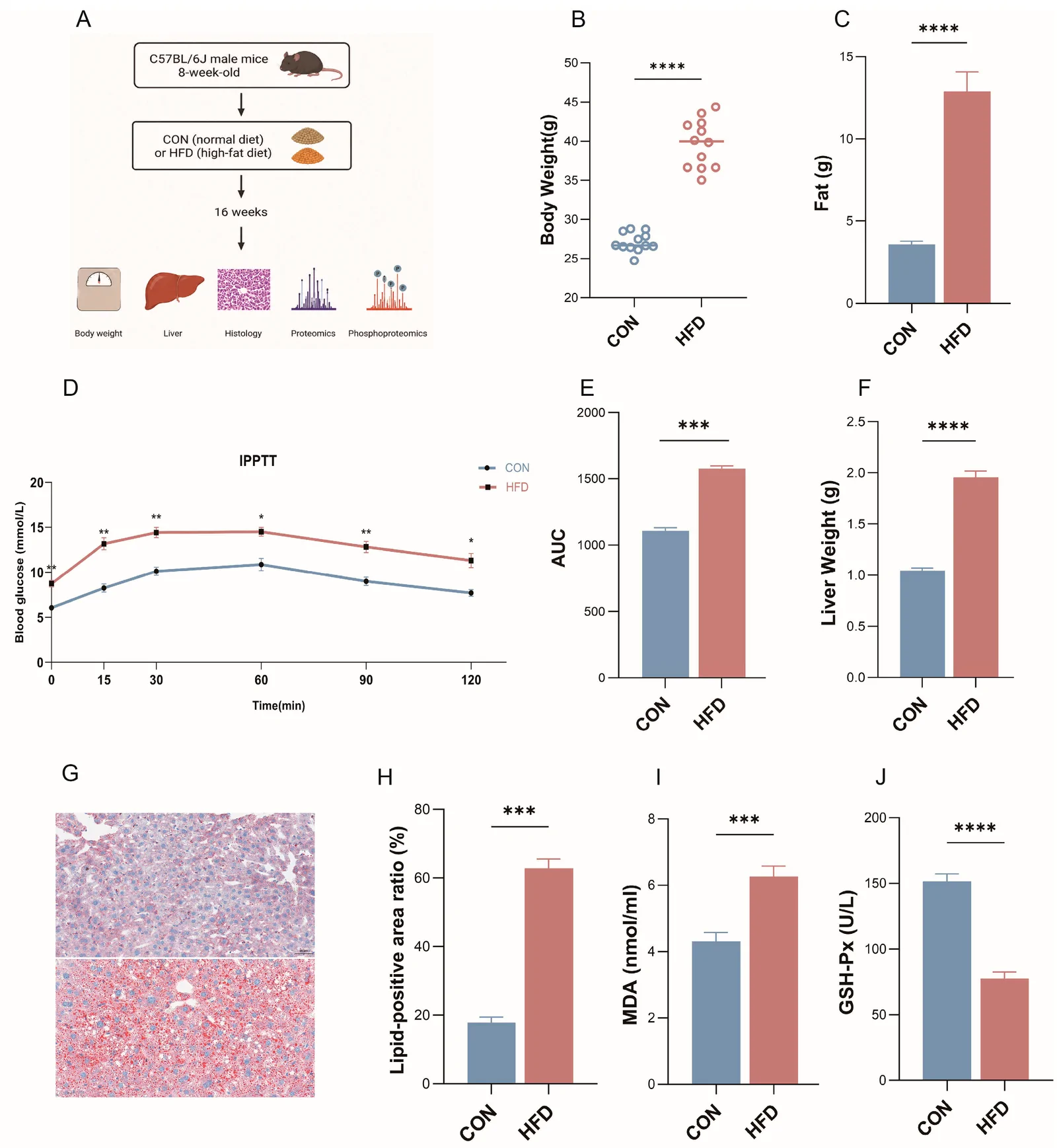
Long-term high-fat diet feeding induces obesity, impaired glucose homeostasis, hepatic lipid accumulation, and systemic oxidative stress in mice. (**A**) Schematic representation of the experimental design. Eight-week-old male C57BL/6J mice were fed either a control diet (CON) or a high-fat diet (HFD) for 16 weeks, followed by physiological assessment, liver histology, proteomic analysis, and phosphoproteomic analysis. (**B**) Final body weight. (**C**) Fat mass. (**D**) Blood glucose concentrations during the intraperitoneal pyruvate tolerance test (IPPTT). (**E**) Total area under the blood glucose curve (AUC) during the IPPTT. (**F**) Liver weight. (**G**) Representative images of Oil Red O-stained liver sections from the CON group (upper) and HFD group (lower). (**H**) Quantification of the Oil Red O-positive area. (**I**) Serum malondialdehyde (MDA) level. (**J**) Serum glutathione peroxidase (GSH-Px) activity. (**B**,**C**,**F**) *n* = 12 mice per group; (**D**,**E**,**I**,**J**) *n* = 6 mice per group; (**G**,**H**) *n* = 3 mice per group. Data are presented as the mean ± SEM. The IPPTT glucose curve was analyzed using two-way repeated-measures ANOVA with Geisser–Greenhouse correction, followed by Šídák’s multiple-comparisons test for between-group comparisons at each time point. The remaining quantitative variables were analyzed using two-tailed unpaired *t*-tests. Significance symbols in (**D**) indicate comparisons between the HFD and CON groups at the corresponding time points, whereas those in the other panels indicate overall comparisons between the HFD and CON groups. * *p* < 0.05, ** *p* < 0.01, *** *p* < 0.001, and **** *p* < 0.0001.

To evaluate the effects of long-term HFD consumption on systemic metabolic status and hepatic lipid homeostasis, 8-week-old male C57BL/6J mice were fed either a normal chow diet or an HFD for 16 weeks, followed by phenotypic, histological, proteomic, and phosphoproteomic analyses (Figure 1A). At the end of the feeding period, body weight and fat mass were significantly higher in the HFD group than in the CON group (both *p* < 0.0001; Figure 1B,C), indicating that long-term HFD consumption induced marked weight gain and fat accumulation.

During the intraperitoneal pyruvate tolerance test, blood glucose levels changed significantly over time in both groups and were consistently higher in the HFD group than in the CON group. The main effects of time and dietary treatment were statistically significant (both *p* < 0.0001), whereas the time × dietary treatment interaction was not significant (*p* = 0.8368; Figure 1D). After adjustment using Šídák’s multiple-comparisons test, blood glucose levels were higher in the HFD group than in the CON group at all measured time points (*p* < 0.05 or *p* < 0.01). The area under the blood glucose curve was also significantly increased in the HFD group (*p* < 0.001; Figure 1E). However, after correction for each mouse’s baseline glucose, the iAUC did not differ significantly between the CON and HFD groups (Figure S1).

Compared with the CON group, liver weight was significantly increased in the HFD group (*p* < 0.0001; Figure 1F). Oil Red O staining revealed only a small number of lipid droplets in the livers of CON mice, whereas extensive and dense lipid deposition was observed in the HFD group (Figure 1G). Quantitative analysis further showed that the proportion of Oil Red O-positive area was significantly higher in the HFD group (*p* < 0.001; Figure 1H). In addition, serum malondialdehyde (MDA) levels were significantly elevated (*p* < 0.001; Figure 1I), whereas serum glutathione peroxidase (GSH-Px) activity was significantly reduced (*p* < 0.0001; Figure 1J), indicating increased lipid peroxidation accompanied by impaired antioxidant defense. Collectively, 16 weeks of HFD feeding successfully established a mouse model characterized by obesity, impaired glucose homeostasis, hepatic lipid accumulation, and increased oxidative stress, providing a reliable experimental basis for subsequent analyses of hepatic protein abundance and phosphorylation-mediated regulation.

### 3.2. Identification of the Mouse Liver Proteome and Analysis of Differentially Expressed Proteins

**Figure 2:**
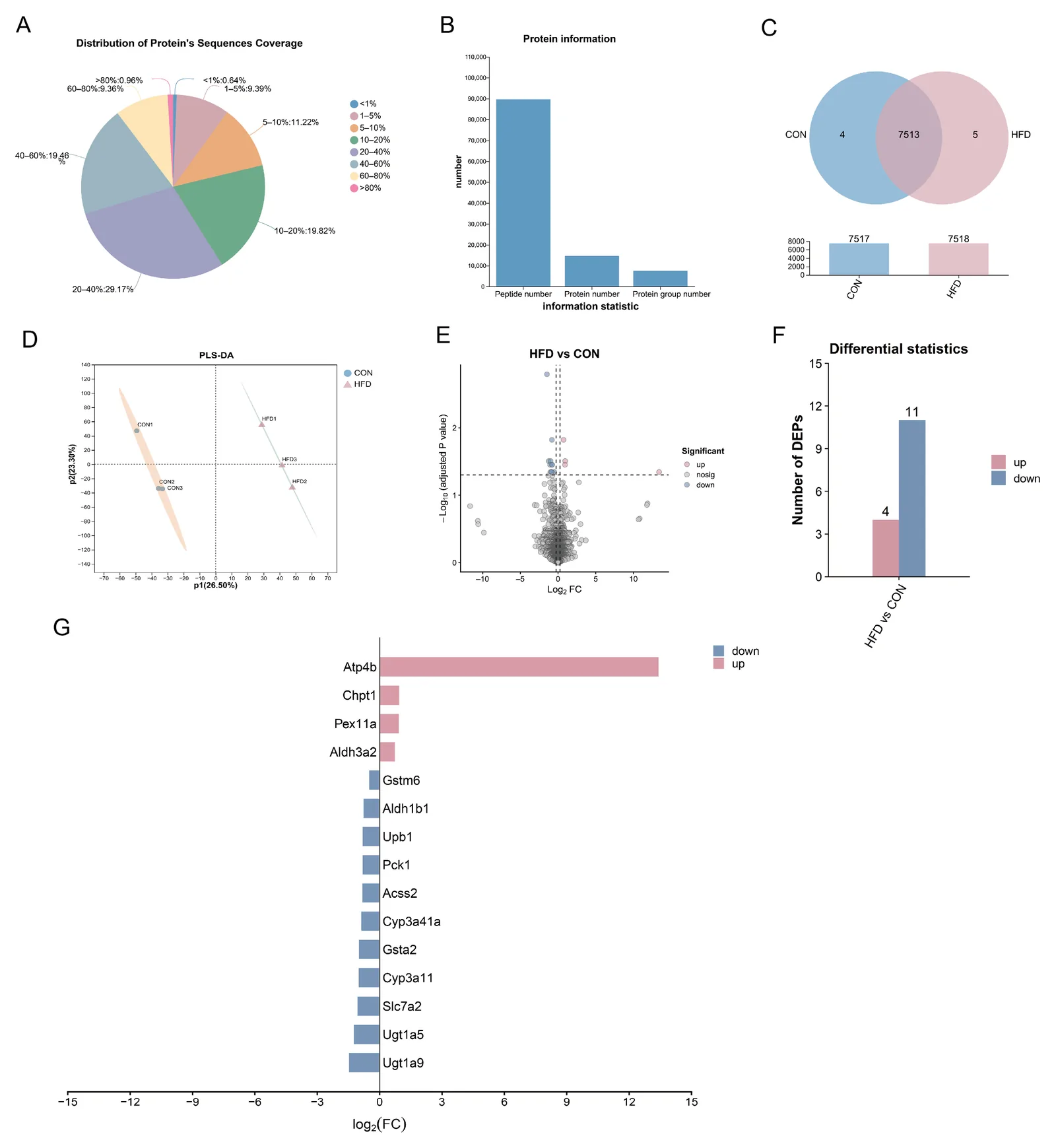
Overview of liver proteome identification and differentially expressed protein analysis in mice. (**A**) Distribution of sequence coverage among the identified proteins. (**B**) Summary of proteomic identification information, including the numbers of identified peptides, proteins, and protein groups. (**C**) Venn diagram showing the shared and group-specific proteins identified in the CON and HFD groups. (**D**) Partial least squares discriminant analysis (PLS-DA) score plot based on the protein abundance profiles. (**E**) Volcano plot of differentially expressed proteins between the HFD and CON groups; red, blue, and gray indicate significantly upregulated, significantly downregulated, and nonsignificantly changed proteins, respectively. (**F**) Numbers of significantly upregulated and downregulated proteins in the HFD group relative to the CON group. (**G**) Log_2_ fold changes (log_2_FC) of all significantly upregulated and downregulated proteins. Red and blue also indicate upregulation and downregulation, respectively, in panels (**F**) and (**G**). Differential abundance testing was performed using limma, with BH correction across all tested proteins. Proteins with a BH-adjusted *p* value ≤ 0.05 and either FC ≥ 1.20 or FC ≤ 0.83 were considered differentially expressed. Proteomic analyses were performed using liver samples from three mice per group (*n* = 3 biological replicates per group). CON, normal diet control; HFD, high-fat diet.

To characterize HFD-induced changes in hepatic protein abundance, protein identification results and between-group differences were first examined. The sequence coverage of the identified proteins spanned a broad range and was predominantly distributed between 10% and 60%, indicating that the mass spectrometry data provided good coverage of proteins with varying abundances and sequence characteristics (Figure 2A). The numbers of identified peptides, proteins, and protein groups are summarized in Figure 2B. In total, 7517 and 7518 proteins were identified in the CON and HFD groups, respectively, of which 7513 were shared between the two groups. Four proteins were detected exclusively in the CON group, whereas five were detected exclusively in the HFD group (Figure 2C), indicating comparable overall proteome coverage between the groups.

Partial least squares discriminant analysis (PLS-DA) based on protein abundance profiles showed clear separation between the CON and HFD samples in the score plot, suggesting that 16 weeks of HFD feeding markedly altered the hepatic proteomic profile of mice (Figure 2D). A volcano plot was generated based on the results of limma differential abundance testing with BH correction. Differential analysis identified 15 differentially expressed proteins with a BH-adjusted *p* value ≤ 0.05 and either FC ≥ 1.20 or FC ≤ 0.83, of which 4 showed significantly increased abundance and 11 showed significantly decreased abundance in the HFD group relative to the CON group (Figure 2E,F). The log_2_FC distribution of all 15 differentially expressed proteins further demonstrated that HFD-induced changes in protein abundance occurred in both directions and involved multiple proteins associated with hepatic metabolism and lipid homeostasis (Figure 2G).

### 3.3. Functional Enrichment and Interaction Network Analyses of Differentially Expressed Hepatic Proteins

**Figure 3:**
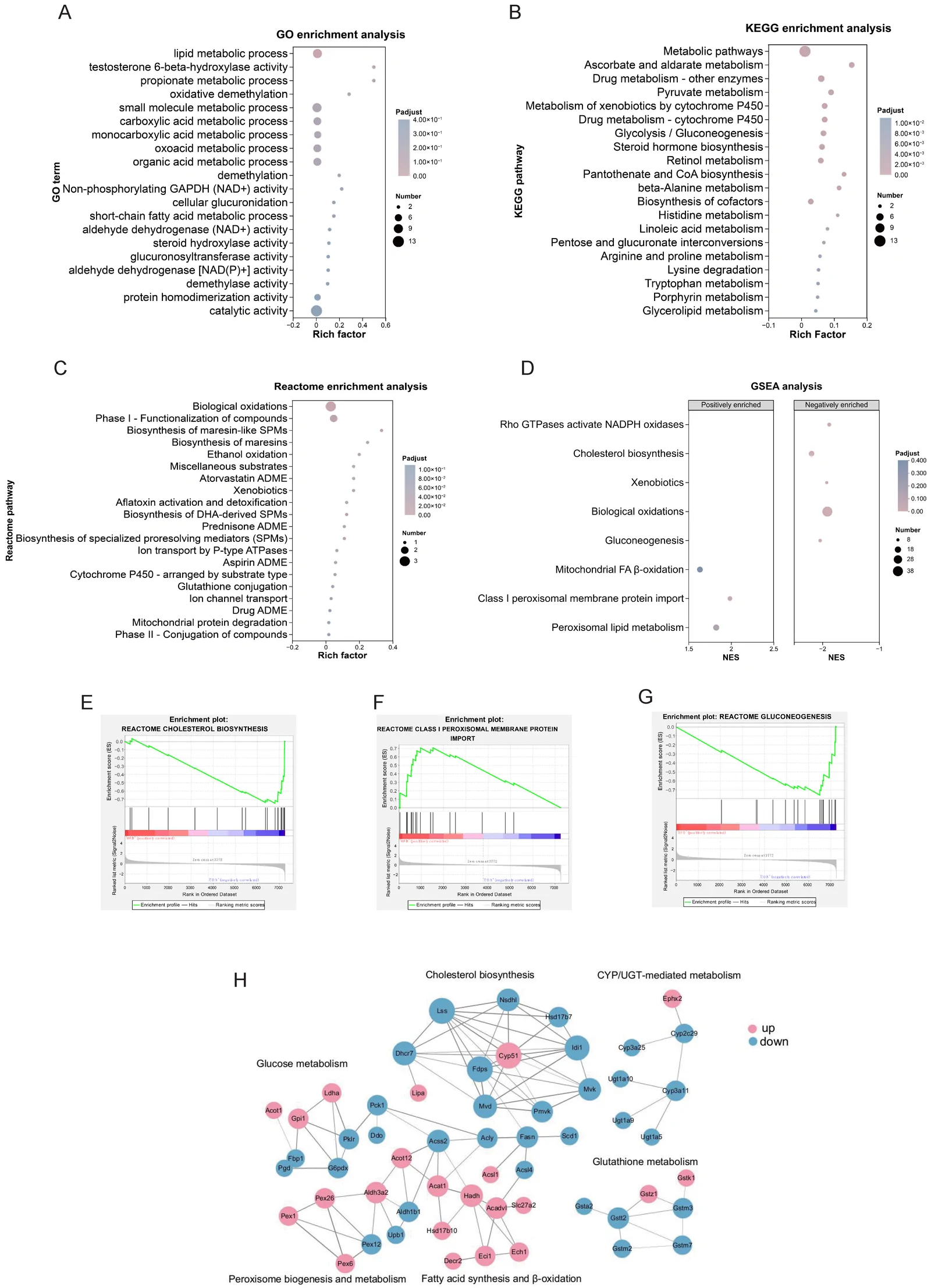
Functional enrichment analyses and protein–protein interaction network of differentially expressed liver proteins. (**A**–**C**) Gene Ontology (GO) (**A**), Kyoto Encyclopedia of Genes and Genomes (KEGG) (**B**), and Reactome pathway (**C**) enrichment analyses of 15 differentially expressed proteins between the HFD and CON groups identified using limma with a BH-adjusted *p* value ≤ 0.05 and either FC ≥ 1.20 or FC ≤ 0.83. The *x*-axis indicates the rich factor, bubble size represents the number of differentially expressed proteins assigned to each term or pathway, and bubble color indicates the adjusted *p* value. (**D**) Summary of gene set enrichment analysis (GSEA), showing representative pathways with positive or negative normalized enrichment scores (NES) in the HFD group relative to the CON group. The *x*-axis indicates the normalized enrichment score (NES), bubble size represents the number of proteins in each pathway, and bubble color indicates the FDR *q* value. (**E**–**G**) Representative GSEA enrichment plots for cholesterol biosynthesis (**E**), class I peroxisomal membrane protein import (**F**), and gluconeogenesis (**G**). For GSEA (**D**–**G**), statistical significance was defined as an FDR *q* value < 0.05. (**H**) Exploratory protein–protein interaction (PPI) network based on the original protein set selected using a two-sided Student’s *t*-test with an unadjusted *p* value ≤ 0.05 and either FC ≥ 1.20 or FC ≤ 0.83, constructed using STRING and visualized in Cytoscape. Node colors indicate the direction of protein abundance changes in the HFD group relative to the CON group, with red and blue nodes representing upregulated and downregulated proteins, respectively. Node size represents degree, and edge width represents the STRING combined interaction score. CON, normal diet control; HFD, high-fat diet; NES, normalized enrichment score.

To investigate the biological processes associated with HFD-induced alterations in hepatic protein abundance, functional enrichment analysis was performed on the 15 differentially expressed proteins identified using limma with BH correction. Gene Ontology (GO) analysis showed that these proteins were mapped to terms including lipid metabolic process, small molecule metabolic process, monocarboxylic acid metabolic process, organic acid metabolic process, oxidative demethylation, and cellular glucuronidation (Figure 3A). Kyoto Encyclopedia of Genes and Genomes (KEGG) analysis mapped these proteins to pathways including Steroid hormone biosynthesis, Glycerolipid metabolism, Glycolysis/Gluconeogenesis, Metabolism of xenobiotics by cytochrome P450, Pyruvate metabolism, and Retinol metabolism (Figure 3B). Reactome analysis further mapped these proteins to pathways including Biological oxidations, Phase I—Functionalization of compounds, Xenobiotics, Glutathione conjugation, and Phase II—Conjugation of compounds (Figure 3C). Together, these analyses identified functional categories related to hepatic metabolism, including lipid metabolism, biological oxidation, and xenobiotic metabolism.

To minimize the loss of information associated with threshold-based selection of differentially expressed proteins, GSEA was further performed using all quantified proteins. Class I peroxisomal membrane protein import was significantly positively enriched in the HFD group, whereas Cholesterol biosynthesis, Gluconeogenesis, Biological oxidations, and Xenobiotics were significantly negatively enriched (all FDR *q* < 0.05; Figure 3D). Peroxisomal lipid metabolism and Mitochondrial fatty acid β-oxidation showed positive NES values but did not meet the significance threshold. Representative enrichment plots illustrate significant negative enrichment of Cholesterol biosynthesis and Gluconeogenesis and significant positive enrichment of Class I peroxisomal membrane protein import (Figure 3E–G).

Subsequent exploratory protein–protein interaction (PPI) analysis, based on the original protein set selected using a two-sided Student’s *t*-test with an unadjusted *p* value ≤ 0.05 and either FC ≥ 1.20 or FC ≤ 0.83, showed that the selected proteins formed several closely interconnected functional modules, primarily involving Glucose metabolism, Cholesterol biosynthesis, Fatty acid synthesis and β-oxidation, Peroxisome biogenesis and metabolism, CYP/UGT-mediated metabolism, and Glutathione metabolism (Figure 3H). Interactions were also observed among these modules, providing an exploratory view of connections among proteins associated with lipid synthesis and degradation, peroxisomal function, and redox metabolism.

### 3.4. Identification of the Mouse Liver Phosphoproteome and Analysis of Differentially Phosphorylated Sites

**Figure 4:**
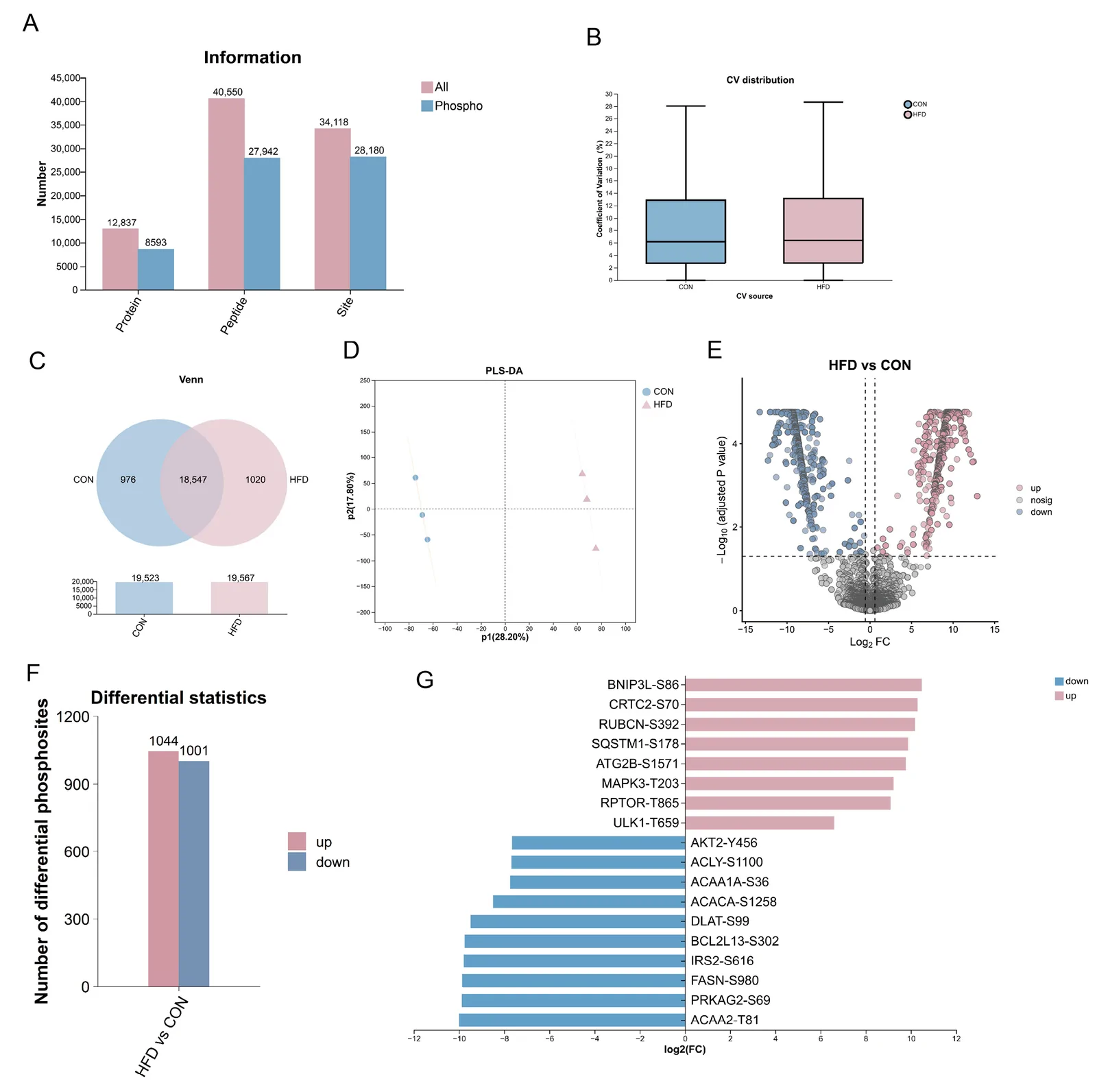
Overview of liver phosphoproteomic identification and differential phosphorylation analysis in mice. (**A**) Summary of phosphoproteomic database search results, showing the numbers of proteins, peptides, and phosphorylation sites in the complete identification dataset (All) and the phosphorylated subset (Phospho). (**B**) Distribution of coefficients of variation (CVs) for the quantitative data in the CON and HFD groups. (**C**) Venn diagram showing the shared and group-specific phosphorylation sites detected in the CON and HFD groups. (**D**) Partial least squares discriminant analysis (PLS-DA) score plot based on phosphosite abundance profiles. (**E**) Volcano plot of differentially phosphorylated sites between the HFD and CON groups; red, blue, and gray indicate significantly upregulated, significantly downregulated, and nonsignificantly changed phosphorylation sites, respectively. (**F**) Numbers of significantly upregulated and downregulated phosphorylation sites in the HFD group relative to the CON group. (**G**) Log_2_ fold changes (log_2_FC) of representative upregulated and downregulated phosphorylation sites. Red and blue also indicate upregulation and downregulation, respectively, in panels (**F**) and (**G**). Differential phosphosite abundance testing was performed using limma, with BH correction across all tested phosphorylation sites. Sites with a BH-adjusted *p* value ≤ 0.05 and either FC ≥ 1.50 or FC ≤ 0.67 were considered differentially phosphorylated. Phosphoproteomic analyses were performed using liver samples from three mice per group (*n* = 3 biological replicates per group). CON, normal diet control; HFD, high-fat diet; CV, coefficient of variation; PLS-DA, partial least squares discriminant analysis.

To further investigate the effects of HFD feeding on hepatic protein post-translational modifications, phosphoproteomic analysis was performed on liver tissues from the CON and HFD groups. Database searching identified a total of 12,837 proteins, 40,550 peptides, and 34,118 sites. Among these, 8593 phosphoproteins, 27,942 phosphopeptides, and 28,180 phosphorylation sites were identified (Figure 4A). The coefficient of variation (CV) distributions of the quantitative data were comparable between the two groups, with similar medians and interquartile ranges, indicating broadly consistent levels of within-group variability in the CON and HFD samples (Figure 4B).

A total of 19,523 and 19,567 phosphorylation sites were detected in the CON and HFD groups, respectively. Of these, 18,547 sites were shared between the two groups, whereas 976 and 1020 sites were detected exclusively in the CON and HFD groups, respectively (Figure 4C). The PLS-DA model based on phosphorylation-site abundance profiles showed clear separation between the two groups along the first component, consistent with differences in hepatic phosphosite abundance profiles between the CON and HFD groups (Figure 4D).

Using limma with a BH-adjusted *p* value ≤ 0.05 and either FC ≥ 1.50 or FC ≤ 0.67 as the selection criteria, 2045 differentially phosphorylated sites were identified. Among these, 1044 sites showed increased phosphosite abundance and 1001 showed decreased phosphosite abundance in the HFD group relative to the CON group (Figure 4E,F). Among the representative differentially phosphorylated sites, phosphosite abundance was increased at BNIP3L-S86, CRTC2-S70, RUBCN-S392, SQSTM1-S178, ATG2B-S1571, MAPK3-T203, RPTOR-T865, and ULK1-T659, whereas it was decreased at AKT2-Y456, ACLY-S1100, ACAA1A-S36, ACACA-S1258, DLAT-S99, BCL2L13-S302, IRS2-S616, FASN-S980, PRKAG2-S69, and ACAA2-T81 (Figure 4G). The proteins harboring these sites are involved in lipid and energy metabolism, insulin signaling, mTOR regulation, and autophagy, indicating that HFD feeding was accompanied by extensive alterations in phosphosite abundance in addition to changes in hepatic protein abundance. Western blot analysis showed a lower hepatic p-AKT/AKT ratio in the HFD group than in the CON group (*p* < 0.05; Figure S2).

To assess phosphosite abundance changes after accounting for parent-protein abundance, an additional analysis was performed on 7565 phosphosites matched to quantified parent proteins by exact accession IDs. Following sample-wise adjustment and limma analysis with BH correction, 703 phosphosites showed significant differences in parent-protein-adjusted abundance between the HFD and CON groups, with 363 higher and 340 lower in the HFD group (Table S3). Eleven representative sites shown in Figure 4G retained significant differences in the same direction after parent-protein adjustment. These included higher adjusted abundances at BNIP3L-S86, SQSTM1-S178, ATG2B-S1571, and MAPK3-T203, and lower adjusted abundances at AKT2-Y456, ACLY-S1100, ACAA1A-S36, ACACA-S1258, DLAT-S99, BCL2L13-S302, and FASN-S980 in the HFD group relative to the CON group (Table S3).

### 3.5. Functional Enrichment and Interaction Network Analyses of Differentially Phosphorylated Hepatic Proteins

**Figure 5:**
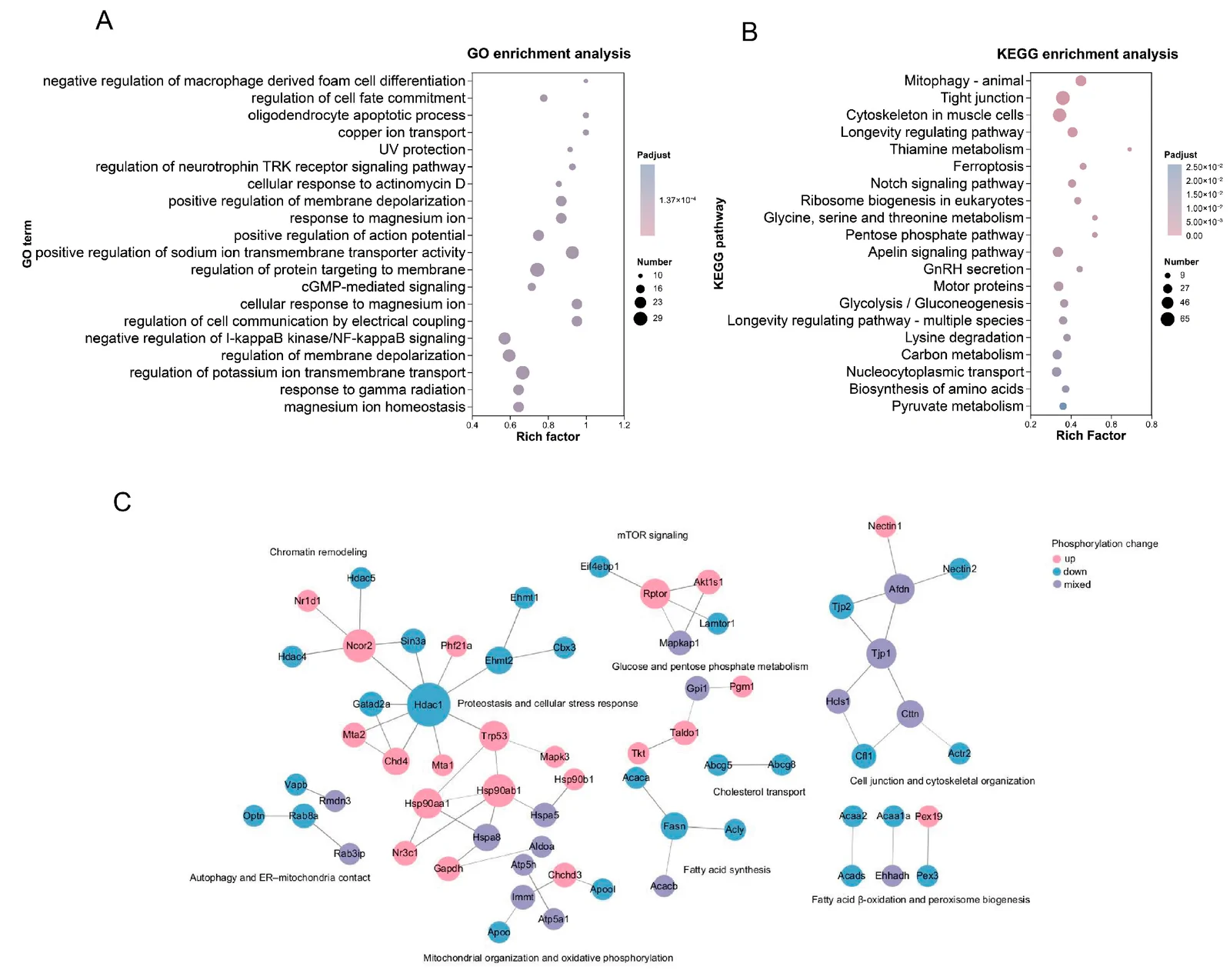
Functional enrichment analyses and protein–protein interaction network of differentially phosphorylated liver proteins. (**A**,**B**) Gene Ontology (GO) (**A**) and Kyoto Encyclopedia of Genes and Genomes (KEGG) (**B**) enrichment analyses of proteins harboring the 2045 differentially abundant phosphosites identified using BH-adjusted *p*-value and fold-change criteria. The *x*-axis indicates the rich factor, bubble size represents the number of proteins harboring differentially phosphorylated sites assigned to each term or pathway, and bubble color indicates the BH-adjusted *p* value. Enrichment was considered significant at a BH-adjusted *p* value < 0.05. (**C**) Exploratory protein–protein interaction (PPI) network of differentially phosphorylated proteins constructed using STRING and visualized in Cytoscape, showing the major functional modules. Node colors indicate the direction of significant phosphosite abundance changes in the HFD group relative to the CON group: red nodes represent proteins containing only significantly upregulated phosphosites, blue nodes represent proteins containing only significantly downregulated phosphosites, and purple nodes represent proteins containing both significantly upregulated and downregulated phosphosites. Node size represents degree, and edge width represents the STRING combined interaction score. CON, normal diet control; HFD, high-fat diet; PPI, protein–protein interaction.

To investigate the biological processes potentially involving proteins harboring differentially phosphorylated sites, GO and KEGG enrichment analyses were performed. GO analysis showed that these proteins were significantly enriched in terms including positive regulation of sodium ion transmembrane transporter activity, positive regulation of membrane depolarization, and magnesium ion homeostasis. In addition, cGMP-mediated signaling and regulation of cell communication by electrical coupling were significantly enriched (Figure 5A). These findings indicate that HFD-induced phosphosite abundance alterations involve multiple biological processes related to membrane transport, cellular signaling, and membrane organization.

KEGG analysis showed that proteins harboring differentially phosphorylated sites were significantly enriched in pathways including Mitophagy–animal, Glycolysis/Gluconeogenesis, Pentose phosphate pathway, Biosynthesis of amino acids, Lysine degradation, and Glycine, serine and threonine metabolism. Enrichment was also observed in Tight junction, Cytoskeleton in muscle cells and Notch signaling pathway (Figure 5B). Thus, HFD-induced alterations in phosphosite abundance were associated not only with hepatic nutrient metabolism but also with organelle homeostasis, intercellular junctions, and signal transduction.

PPI network analysis further showed that the differentially phosphorylated proteins formed several functional modules, with connections observed between some modules (Figure 5C). These modules primarily included mTOR signaling, Autophagy and ER–mitochondria contact, Mitochondrial organization and oxidative phosphorylation, Proteostasis and cellular stress response, Fatty acid synthesis, Fatty acid β-oxidation and peroxisome biogenesis, Glucose and pentose phosphate metabolism, Cholesterol transport, Cell junction and cytoskeletal organization, and Chromatin remodeling. Collectively, the enrichment and PPI network analyses indicated that HFD-induced alterations in hepatic phosphosite abundance involved proteins associated with nutrient sensing, lipid and energy metabolism, organelle homeostasis, and cellular structural regulation.

## 4. Discussion

By integrating whole-animal phenotyping with hepatic proteomic and phosphoproteomic analyses, this study investigated the effects of long-term HFD feeding on the mouse liver at the levels of protein abundance and post-translational modification. Sixteen weeks of HFD feeding significantly increased body weight, fat mass, and liver weight and caused extensive hepatic lipid droplet accumulation. Meanwhile, HFD-fed mice maintained higher blood glucose levels during the IPPTT and exhibited increased serum MDA levels and decreased GSH-Px activity, indicating that this model was characterized by obesity, hepatic lipid accumulation, impaired glucose homeostasis, and systemic redox imbalance. C57BL/6J mice are particularly sensitive to dietary fat loading and are therefore widely used to investigate HFD-associated hepatic metabolic abnormalities [17,18,19]. Previous studies have shown that oxidative stress can emerge early during the development of HFD-induced obesity and insulin resistance and may contribute to the subsequent progression of metabolic dysfunction [20]. Thus, the concurrent presence of obesity, disturbed glucose homeostasis, hepatic lipid accumulation, and systemic redox imbalance provides the phenotypic basis for the proteomic and phosphoproteomic alterations observed in this study.

Proteomic analysis identified 15 differentially expressed proteins, including 4 with increased abundance and 11 with decreased abundance. Functional analyses mapped these proteins to categories related to lipid and small-molecule metabolism, biological oxidation, and xenobiotic metabolism. Although the numbers and overall coverage of proteins identified in the CON and HFD groups were comparable, the two groups were clearly separated by PLS-DA. This separation was consistent with differences in protein abundance profiles, although the supervised score plot alone does not establish the robustness of the group discrimination. The combination of comparable identification coverage and differential abundance results indicates that the detected differences primarily concerned the abundance of shared proteins rather than extensive group-specific protein detection. Previous mouse liver proteomic studies have similarly shown that HFD feeding alters the expression of proteins involved in lipid metabolism, redox regulation, mitochondrial function, and cellular injury [21,22]. In the present study, CHPT1, ALDH3A2, and PEX11A abundance increased, whereas ACSS2 abundance decreased. CHPT1 participates in phosphatidylcholine synthesis [23], while ALDH3A2 catalyzes fatty aldehyde oxidation and contributes to cellular responses to fatty acid-associated stress [24]. ACSS2 converts acetate to acetyl-CoA and participates in lipid metabolism [25], and its decreased abundance may reflect an adjustment in hepatic utilization of acetate-derived carbon. PEX11A participates in peroxisome elongation and division [26], and its increased abundance was consistent with the positive enrichment patterns involving peroxisomal lipid metabolism and membrane protein import, supporting a role for peroxisomes in hepatic adaptation to chronic lipid loading. In addition, CYP3A11 and CYP3A41A, which participate in oxidative xenobiotic metabolism, and UGT1A5, UGT1A9, GSTA2, and GSTM6, which participate in glucuronide or glutathione conjugation, all showed decreased abundance, indicating a consistent decline in several enzymes involved in these processes at the protein abundance level. Collectively, long-term HFD feeding was accompanied by distinct changes in proteins involved in hepatic lipid handling and xenobiotic metabolism, reflecting multiple aspects of the hepatic metabolic response to sustained lipid loading.

A notable finding was the positive normalized enrichment scores for mitochondrial fatty acid β-oxidation, peroxisomal lipid metabolism, and Class I peroxisomal membrane protein import in the HFD group. The coexistence of hepatic lipid accumulation and these protein abundance patterns is not contradictory. Under conditions of sustained fatty acid influx, increased abundance of proteins involved in mitochondrial and peroxisomal oxidation may represent a compensatory response by hepatocytes to limit lipid accumulation [27,28]. PPARα is a key transcriptional regulator of this adaptive response, coordinating fatty acid uptake and activation, mitochondrial and peroxisomal β-oxidation, and ketogenesis [29]. PPARα deficiency impairs hepatic fatty acid oxidation and exacerbates hepatic lipid accumulation under high-fat conditions [30], whereas maintaining PPARα stability in hepatocytes helps alleviate HFD-induced hepatic lipid accumulation [31,32]. Compensatory increases in hepatic mitochondrial respiratory capacity have also been observed in humans with simple hepatic steatosis, whereas this adaptation is progressively lost during the development of steatohepatitis [33].

Adaptation of fatty acid oxidation may also impose a redox burden. A sustained increase in fatty acid supply can increase the reducing load on the mitochondrial electron transport chain. When electron transfer is impaired, electron leakage and reactive oxygen species (ROS) production may increase [34,35,36]. Previous studies have shown that 16 weeks of HFD feeding disrupts hepatic oxygen gradients and mitochondrial function and is accompanied by increased CYP2E1 abundance and oxidative stress [37]. In the present study, the abundance of several proteins involved in glutathione conjugation and CYP/UGT-mediated metabolism decreased, while serum MDA levels increased and GSH-Px activity decreased, indicating that HFD-induced lipid accumulation was accompanied by systemic redox imbalance.

The positive GSEA scores for peroxisomal lipid metabolism and Class I peroxisomal membrane protein import also associated HFD feeding with changes in proteins involved in peroxisomal lipid handling and membrane protein import. These abundance patterns may reflect peroxisomal adaptation to sustained lipid loading [38,39]. GSEA further revealed negative normalized enrichment scores for cholesterol biosynthesis, gluconeogenesis, biological oxidation, and xenobiotic metabolism in the HFD group. Reduced abundance of cholesterol biosynthesis-related proteins does not necessarily indicate a lower hepatic lipid burden because hepatic triglycerides are derived not only from de novo lipogenesis but also from dietary lipids and free fatty acids released from adipose tissue [40,41]. Concurrently, increased intracellular cholesterol can restrict the transport of SREBP2 from the endoplasmic reticulum to the Golgi apparatus and its subsequent proteolytic activation through SCAP–INSIG-mediated feedback, thereby suppressing the expression of cholesterol biosynthesis-related genes and proteins [42]. Therefore, the negative enrichment of this pathway may reflect feedback regulation in response to increased lipid availability.

Notably, although gluconeogenesis-related proteins showed an overall downward trend, baseline blood glucose and the total IPPTT area under the curve remained significantly elevated in the HFD group, whereas baseline-adjusted iAUC did not differ significantly between groups. This distinction indicates that the higher total AUC partly reflected elevated baseline glycemia rather than establishing a greater glucose excursion above baseline. Metabolic flux is also regulated at multiple levels, including enzyme activity, substrate availability, the hormonal environment, and interorgan metabolic interactions [43]. Thus, the protein abundance changes and the glycemic phenotype describe different aspects of metabolic regulation. Together, the IPPTT findings support disturbed glucose homeostasis without establishing increased hepatic gluconeogenic flux [44].

The phosphoproteomic results further demonstrated that HFD-induced alterations were also evident at the phosphosite abundance level. A total of 2045 differentially phosphorylated sites were identified, including 1044 sites with increased abundance and 1001 with decreased abundance. This distribution indicates that HFD feeding was accompanied by bidirectional changes in hepatic phosphosite abundance rather than a uniform increase or decrease. Because phosphosite abundance can also be affected by changes in the corresponding parent protein abundance, these results should not be directly equated with changes in phosphorylation occupancy. A complementary parent-protein-adjusted analysis was therefore performed to assess this contribution. The adjusted results provide additional site-level support for changes involving autophagy- and mitophagy-associated proteins (BNIP3L, SQSTM1, ATG2B, and BCL2L13), enzymes involved in lipid and energy metabolism (ACACA, FASN, ACLY, ACAA1A, and DLAT), and signaling proteins (AKT2 and MAPK3). Krahmer et al. similarly reported widespread alterations in protein localization and phosphorylation states across lipid droplets, the endoplasmic reticulum, mitochondria, and peroxisomes in diet-induced hepatic steatosis, supporting the relevance of organelle organization to hepatic adaptation to lipid loading [45]. Based on the PPI network and manual functional annotation, proteins harboring differentially phosphorylated sites were primarily associated with mTOR signaling, autophagy, mitochondrial organization, oxidative phosphorylation, endoplasmic reticulum–mitochondria contact, peroxisome biogenesis, and cholesterol transport.

Among the differentially phosphorylated sites, phosphosite abundance was decreased at IRS2-S616 and AKT2-Y456 but increased at MAPK3-T203, identifying site-level differences in proteins associated with insulin-related signaling and the MAPK pathway. However, these findings require cautious interpretation. AKT2-Y456 is not a canonical site commonly used to assess AKT2 activity; therefore, its reduced phosphosite abundance cannot be directly interpreted as decreased AKT2 activity [46]. The reduced p-AKT (Ser473)/AKT ratio further supports altered hepatic AKT signaling following long-term HFD feeding (Figure S2). Furthermore, IRS1/2 proteins contain numerous serine/threonine phosphorylation sites, and different sites may exert inhibitory, stimulatory, or context-dependent effects on protein stability, subcellular localization, and insulin signaling. The site-specific function of IRS2-S616 requires further investigation [47]. Similarly, increased abundance at MAPK3-T203 alone does not establish ERK activation. This interpretation requires assessment of the relevant activating phosphorylation sites and direct validation of ERK1/2 signaling [48].

Autophagy and mitophagy represent another important mechanistic clue emerging from the phosphoproteomic findings. KEGG analysis showed significant enrichment of Mitophagy–animal, whereas the PPI network contained modules associated with mTOR signaling, autophagy, endoplasmic reticulum–mitochondria contact, and mitochondrial organization. Concurrently, altered phosphosite abundance was detected in ULK1, RPTOR, SQSTM1, BNIP3L, RUBCN, ATG2B, and BCL2L13. Autophagy in hepatocytes contributes to lipid droplet degradation and fatty acid mobilization, whereas impaired autophagy promotes hepatic lipid accumulation [49]. Obesity-associated defects in hepatic autophagy can also exacerbate endoplasmic reticulum stress and insulin resistance [13]. At the signaling level, AMPK and mTORC1 coordinate nutrient status with autophagy initiation by phosphorylating ULK1 at distinct sites [11]. Increased RUBCN abundance or activity can inhibit autophagosome maturation and promote lipid accumulation and hepatocellular injury in experimental fatty liver disease [50]. HFD feeding increased phosphosite abundance at MAPK3-T203, RPTOR-T865, and ULK1-T659, suggesting alterations in regulatory processes associated with ERK signaling, mTORC1-mediated nutrient sensing, and autophagy initiation.

Differentially phosphorylated sites were also detected in enzymes involved in fatty acid synthesis or β-oxidation, including ACACA, ACACB, FASN, ACLY, ACAA1A, and ACAA2. These findings suggest that, in addition to altering protein abundance, HFD feeding may regulate the functional state of metabolic enzymes through post-translational modifications. However, increased phosphorylation does not necessarily indicate enhanced enzymatic activity, as its functional consequences depend on the specific site and upstream kinase involved [51]. In particular, PRKAG2 is the γ2 regulatory subunit of the AMPK complex, and phosphorylation at PRKAG2-S69 cannot substitute for phosphorylation at AMPKα-T172 as a readout of AMPK activity [52]. The functions of sites such as ACACA-S1258 and FASN-S980 also remain unclear. These sites should therefore be regarded as candidate targets for subsequent mechanistic validation rather than definitive evidence of functional changes.

Beyond metabolic processes, proteins harboring differentially phosphorylated sites were also associated with ion transport, cytoskeletal organization, cell junctions, and chromatin regulation, indicating that the effects of HFD feeding were not restricted to metabolic enzymes. Because these findings were derived primarily from enrichment analyses, their specific biological significance requires further investigation in relevant cell types and functional experiments.

In summary, long-term HFD feeding not only induced obesity, impaired glucose homeostasis, and hepatic lipid accumulation but was also accompanied by differences in hepatic protein abundance and extensive bidirectional changes in phosphosite abundance. The proteomic findings associated HFD feeding with lipid and small-molecule metabolism, biological oxidation, and xenobiotic metabolism, while complementary GSEA identified abundance patterns involving fatty acid oxidation and peroxisomal processes. The phosphoproteomic findings additionally involved insulin-related signaling, nutrient sensing, proteins associated with mTOR–ULK1 regulation, autophagy, and organelle homeostasis. These results provide candidate molecular insights into hepatic adaptation and dysregulation under chronic lipid loading; however, the functions of key phosphorylation sites and their causal relationships with the observed metabolic phenotypes require further validation. Collectively, these data provide a basis for identifying critical regulatory sites and conducting targeted mechanistic studies.

## 5. Conclusions

Sixteen weeks of HFD feeding induced obesity, impaired glucose homeostasis, and hepatic lipid accumulation in mice, accompanied by increased serum MDA levels and reduced GSH-Px activity. Proteomic analysis identified 15 differentially expressed proteins, primarily associated with lipid and small-molecule metabolism, biological oxidation, and xenobiotic metabolism. Alterations in proteins associated with fatty acid β-oxidation and peroxisomal function further characterized the hepatic response to sustained lipid loading. Phosphoproteomic analysis identified 2045 differentially phosphorylated sites, including sites on the insulin signaling-related proteins IRS2 and AKT2, the nutrient-sensing and autophagy-related proteins RPTOR and ULK1, and lipid metabolic enzymes such as ACACA and FASN. Together, these findings link HFD-induced hepatic steatosis to changes in metabolic protein abundance and phosphosite abundance involving nutrient sensing, lipid metabolism, and organelle homeostasis. The identified proteins and sites provide candidates for functional studies to establish their roles in hepatic steatosis.

## Data Availability

The original contributions presented in this study are included in the article and its Supplementary Materials. Further inquiries can be directed to the corresponding author.

## Supplementary Materials

The following supporting information can be downloaded at: https://www.mdpi.com/article/10.3390/nu18213587/s1, Figure S1: Baseline-adjusted incremental glucose area under the curve during the intraperitoneal pyruvate tolerance test; Figure S2: Hepatic AKT phosphorylation in CON and HFD mice; Table S1: Complete differential protein abundance analysis between HFD and CON groups; Table S2: Complete differential phosphosite abundance analysis between HFD and CON groups; Table S3: Complete differential phosphosite abundance analysis after adjustment for parent-protein abundance.
