# Supplementary material for: Proteomic and Phosphoproteomic Profiling of High-Fat Diet-Induced Hepatic Steatosis in Mice
Source: Nutrients. 2026 Oct 29;18(21):3587. doi: 10.3390/nu18213587 (PMC13764859; doi:10.3390/nu18213587)
Supplement: Supplementary file 1 [file nutrients-18-03587-s001.zip › Supplementary Figure.pdf]

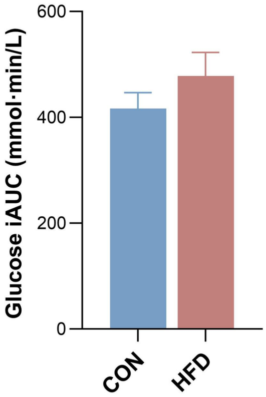

Supplementary Figure S1. Baseline-adjusted incremental glucose area under the curve during the intraperitoneal pyruvate tolerance test. The incremental area under the curve (iAUC) over 0–120 min was calculated for each mouse using the trapezoidal rule after subtracting its own baseline glucose concentration, with areas below baseline retained as negative contributions. Data are presented as mean ± SEM (n = 6 mice/group). Groups were compared using a two-tailed unpaired Student's t-test (p = 0.5288). CON, control diet; HFD, high-fat diet.

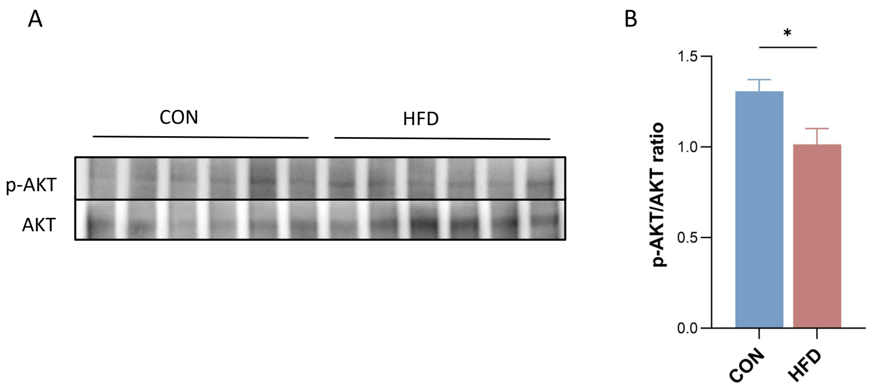

Supplementary Figure S2. Hepatic AKT phosphorylation in CON and HFD mice. (A) Representative Western blots of phosphorylated AKT (Ser473) and total AKT in liver tissues. (B) Quantification of the p-AKT (Ser473)/AKT ratio. Data are presented as the mean ± SEM (n = 6 mice per group). Statistical significance was assessed using a two-tailed unpaired Student's t-test. p <0.05 versus CON. CON, normal diet control; HFD, high-fat diet.
